# Supplementary figures and images for: SMYD3 Modulates the HGF/MET Signaling Pathway in Gastric Cancer
Source: Cells. 2023 Oct 18;12(20):2481. doi: 10.3390/cells12202481 (PMC10605494; doi:10.3390/cells12202481)

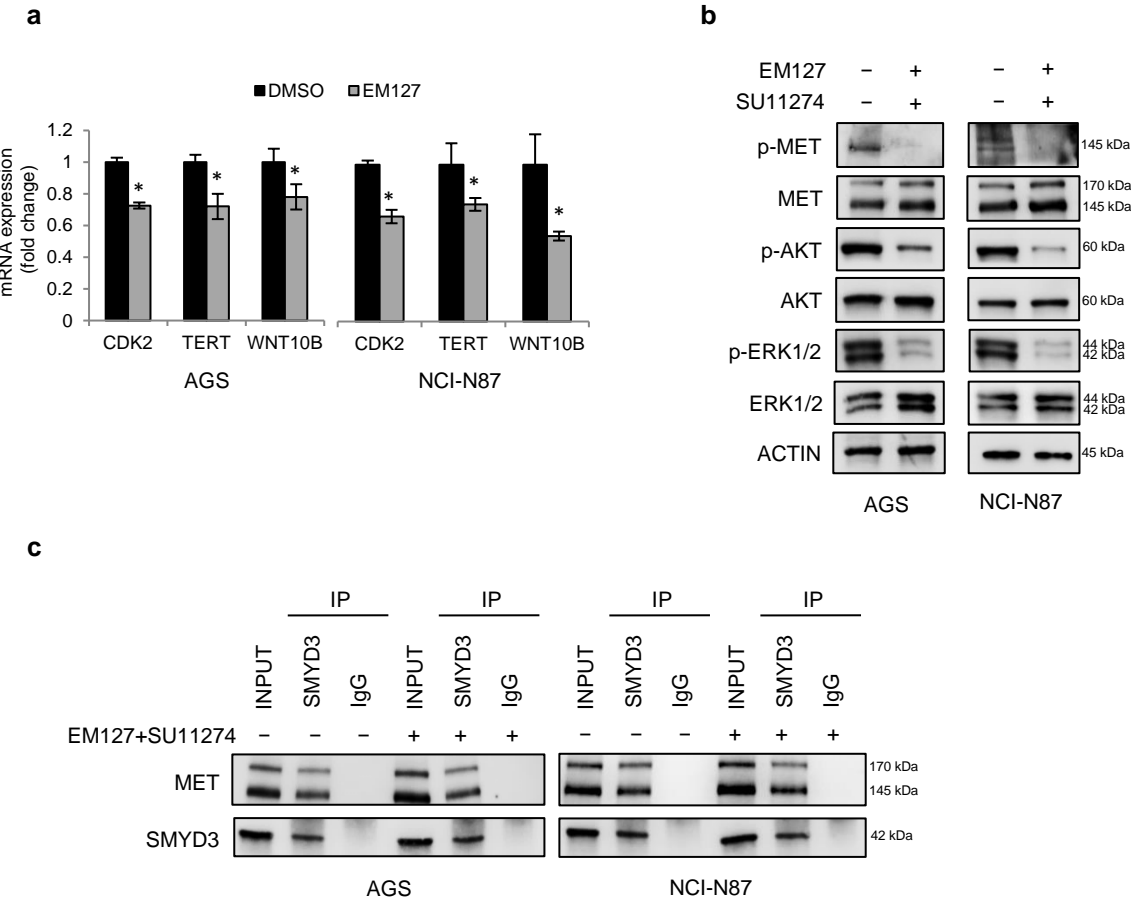

Supplementary Figure S2

a

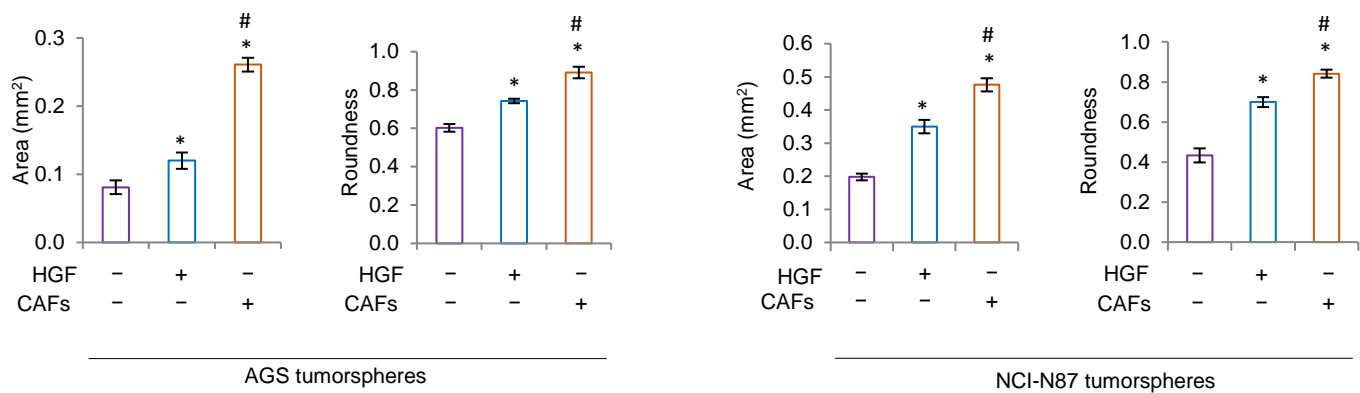

b

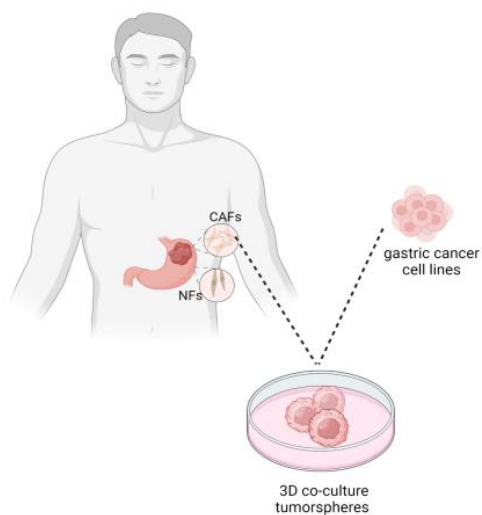

c

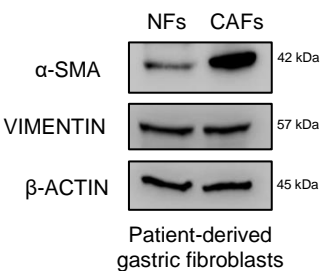

d

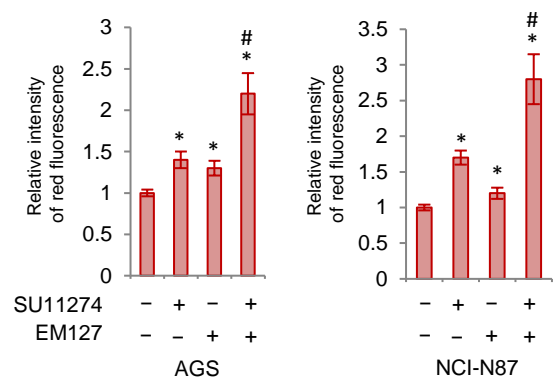

Supplement: Supplementary file 1 [file cells-12-02481-s001.zip › cells-2550389-supplementary.pdf]
